# Supplementary material for: The role of CXCL10 as a biomarker for immunological response among patients with leprosy: a systematic literature review
Source: PeerJ. 2024 Apr 5;12:e17170. doi: 10.7717/peerj.17170 (PMC11000641; doi:10.7717/peerj.17170)
Supplement: Supplemental Information 2 [file peerj-12-17170-s002.docx]

1. **Rationale for conducting the systematic review**

Leprosy is a complicated bacterial infection which manifest in a variety of ways depending on immunological response^1^. Leprosy is linked to CXCL10 expression and investigating CXCL10 role in leprosy’s immunopathogenesis could help diagnosing leprosy apart form using clinical diagnosis criteria^2–6^. Also, CXCL10 had an easily accessible detection method^7–9^. A systematic review on the relationship between leprosy and CXCL10 can help in drawing conclusion of different outcomes from various studies to prove the relevance of CXCL10 as a biomarker in diagnosing leprosy and determining the types of leprosy. This study was conducted because there haven't been any systematic reviews done on CXCL10 and leprosy. Therefore, this one is expected to provide a concise conclusion, especially due to the heterogeneous findings from various studies.

The rationale for conducting the systematic review can be found on pages 2-3 (see the introduction).

1. **The contribution that the systematic review makes to knowledge in light of previously published related reports**

For the first time, the existing experimental research on CXCL10 and leprosy has been methodically synthesized in one comprehensive study. Various studies found CXCL10 to be potential in diagnosing of leprosy particularly in leprosy reactions, determining the types of leprosy, and as indicator of the efficacy of leprosy therapy. However, these findings are not heterogeneous, so further research is needed with larger sample sizes and more diverse methods, taking into consideration other confounding factors in order to establish CXCL10 roles in leprosy.

See the last paragraph of the introduction (page 3), the discussion and conclusion sections for information about the contribution of the systematic review.

1. Nath I. Immunopathogenesis of Leprosy: A Model for T Cell Anergy. *EMJ Dermatology* 2016; 95–101.

2. de Sousa JR, Sotto MN, Quaresma JAS. Leprosy as a complex infection: Breakdown of the Th1 and Th2 immune paradigm in the immunopathogenesis of the disease. *Front Immunol* 2017; 8: 18–21.

3. Ferreira H, Mendes MA, de Mattos Barbosa MG, et al. Potential Role of CXCL10 in Monitoring Response to Treatment in Leprosy Patients. *Front Immunol* 2021; 12: 1–10.

4. Hadi S, Khairunnisa A, Khalifah SN, et al. Skrining Inhibitor NF- κB Combretum indicum dengan Metode Docking. 2021; 18: 157–163.

5. Sharma I, Singh A, Mishra AK, et al. Is CXCL10/CXCR3 axis overexpression a better indicator of leprosy type 1 reaction than inducible nitric oxide synthase? *Indian J Med Res* 2015; 142: 681–689.

6. Hungria EM, Freitas AA, Pontes MAA, et al. Antigen-specific secretion of IFNγ and CXCL10 in whole blood assay detects Mycobacterium leprae infection but does not discriminate asymptomatic infection from symptomatic leprosy. *Diagn Microbiol Infect Dis* 2017; 87: 328–334.

7. Pujiastuti AT, Agusni I, Staf D, et al. Profil Kadar Interferon Gamma Induced Protein 10 Serum pada Pasien Kusta Borderline dengan dan tanpa Reaksi Kusta Tipe 1 ( Profile of Serum Interferon Gamma Induced Protein 10 in Borderline Leprosy Patients with and without Type 1 Leprosy Reaction ). 1.

8. Medeiros MF, Rodrigues MJ, Vital RT, et al. CXCL10, MCP-1, and Other Immunologic Markers Involved in Neural Leprosy. *Appl Immunohistochem Mol Moprhol* 2015; 23: 220–229.

9. Mertaniasih NM, Ananda IGYP, Soedarsono S, et al. Diagnosis Based on Detection of CXCL10 in Urine as Biomarker for The Determining Diagnosis of Active Lung Tuberculosis. *Indones J Trop Infect Dis* 2021; 9: 57.
